# Monitoring Moving Queries inside a Safe Region

**DOI:** 10.1155/2014/630396

**Published:** 2014-02-16

**Authors:** Haidar Al-Khalidi, David Taniar, John Betts, Sultan Alamri

**Affiliations:** Clayton School of Information Technology, Monash University, Clayton, VIC 3800, Australia

## Abstract

With mobile moving range queries, there is a need to recalculate the relevant surrounding objects of interest whenever the query moves. Therefore, monitoring the moving query is very costly. The safe region is one method that has been proposed to minimise the communication and computation cost of continuously monitoring a moving range query. Inside the safe region the set of objects of interest to the query do not change; thus there is no need to update the query while it is inside its safe region. However, when the query leaves its safe region the mobile device has to reevaluate the query, necessitating communication with the server. Knowing when and where the mobile device will leave a safe region is widely known as a difficult problem. To solve this problem, we propose a novel method to monitor the position of the query over time using a linear function based on the direction of the query obtained by periodic monitoring of its position. Periodic monitoring ensures that the query is aware of its location all the time. This method reduces the costs associated with communications in client-server architecture. Computational results show that our method is successful in handling moving query patterns.

## 1. Introduction

With the explosive growth of wireless technology in recent times, devices such as location-aware mobile phones, position locators, and personal navigation systems have become ubiquitous. Because of the increased demand on Location-Based Services (LBS), extensive studies have been conducted to address the continuous monitoring of moving queries.

In this work, we focus on studying the problem of the moving range search (MRS) query, which represents one of the most important types of processing for spatial and geographical information. The MRS query can be defined as follows: given a set of special objects, a query point and a range (radius) find all objects of interest within the radius of the query while the query is moving. Many users' queries such as those in traffic control, online search engines, Geographic Information System (GIS) and wireless sensor networks require an information system with an efficient implementation of MRS query. An example of a moving query using LBS is a car driver who wants to find all petrol stations within a radius of five kilometres from their current location.

A moving query continuously returns sets of objects of interest which extend from the registration of the query to its cancellation. This is called the effective period of the query. Over this time, the query results must be continuously updated even if the query conditions remain unaltered during the *effective period* [1]. To reduce the updating costs when a query is moving continuously, a safe region has been proposed [2–5]. While the moving query remains in the safe region, the set of its objects of interest does not change.

Inside a safe region there is no need for the query to communicate with the server or for the server to update the result list. Thus these two factors have the potential to significantly reduce communication and computational overheads. Safe regions may be simple in shape, that is, circular. However, to increase the size of these regions, larger, irregular safe regions have been proposed [3, 6]. Because the query does not communicate with the server once it has entered the safe region, neither the query nor the server will be aware of when, and from which direction, the query will leave the safe region. As a consequence, the server cannot give the query a new safe region to roam in or update the result list (immediately on the query exiting the current safe region).

In this work, we propose a new approach based on a linear function of time so the query can monitor itself while it is inside the safe region. Our approach supports the concept of random query moves and it is the first to present a monitoring algorithm that continuously monitors moving range queries in mobile navigation. This approach allows the query to roam freely inside its safe region without any involvement from the server. In order to reduce the communication costs between the queries and the server, and also to improve the computational cost, the query will inform the server about its new location only when it leaves its safe region. We present an analysis of the computation and communication costs of our algorithm which shows the benefits of this new algorithm.

The rest of the paper is structured as follows.  Section 2 introduces approaches used by previous researchers to reduce the cost of moving queries. The limitations of these approaches are discussed, and our proposed solution is introduced.  Section 3 presents the theoretical background of (static and moving) range search queries and two types of safe regions (basic and extended).  Section 4 introduces our technique of monitoring the moving range query inside the safe region.  Section 5 introduces the algorithms for creating a safe region and monitoring the query. The experimental results are presented in Section 6.  Section 7 concludes the paper.

## 2. Related Work

A static range search query (or for simplicity range search query) is location-dependent, based on the current user's location [7–10], and assumes a user is stationary. However, the static range query is not adequate for moving users, who may require query updates as their location changes. In response, continuous (moving) queries have been developed. The main disadvantage of a moving query search is the need to maintain up-to-date query results, which increases the wireless communication and query reevaluation costs significantly.

The problem of dealing with data that change continuously due to changes in the position of moving objects was first addressed by Sistla et al. [11]. They identified the importance of the continuous concept in nearest neighbours query, and a new data model was proposed to represent the moving objects in data systems. The function of time was represented by the user's position which changed as time passed, even without an explicit update. Sistla et al. also proposed a query language which enables the specifications of the future queries (i.e., queries that refer to future states of the databases) to be predicted. However, in their study, access or processing methods were not discussed.

In another study, Hu et al. [12] proposed a generic framework to handle continuous queries with safe regions whereby the location updates from mobile clients are further reduced. This study was limited to addressing only a part of the mobility challenge since their conclusions were based on the assumptions that queries are static which might not be possible for real-world applications. Another approach proposed by Cheng et al. [13] was a time-based location update mechanism which was designed to reduce the temporal data inconsistency for the objects relevant to queries. To be able to send location updates more frequently, data objects with significance to the correctness of the query results are required. However, this method had a major limitation: an object would repeatedly and unnecessarily send location updates to the server when it was enclosed by a query region.

Several other studies have also addressed the process of continuous range search queries. In 2013, a study was introduced which adopted the concept of approximation to address the issue of dynamic range search queries in mobile navigation and road networks [14, 15]. In this study, the approximate continuous range search (ACRS) query was proposed, which determines the locations of interest objects as the query moves on a predefined path. The aim of this new query is to reduce the number of critical objects that enter the range search and leave it within a very short time. Consequently, this will give the user enough time to make a decision. The main advantage of this query is that it reduces the number of split points, which reduces the number of location updates. This leads to a decrease in the amount of communication between the mobile device and the database server compared with other query types. However, this method did not solve the problem of the pre-defined path, where the server should be aware of the path that the query will follow throughout its journey.

To track the moving query in a nondefined path (or random path), the concept of path prediction was introduced. Path prediction produces better results and reduces the location update frequency in object tracking while preserving accuracy. Different models for predicting the future position of the moving query have been proposed; however, they have only offered accurate route predictions on a short-term basis.

A simple prediction model was introduced by Jensen et al. to represent an object's future location as a linear function of time which is based on the most recently reported location and velocity of the object. This representation is typically adopted in the context of indexing because it is compact, easy to obtain, and reduces the number of updates compared with updating at constant time intervals [16]. However, this model did not offer accurate predictions beyond the short term and suffered from the *fork dilemma*. In response to that, a more complex, nonlinear prediction model was introduced by Tao et al. [17]. In this model, the recursive motion function (RMF) achieved better predictions by finding a curve that best fits the last few reported locations of the moving object. However, the problem of the fork dilemma was not solved in this model and the model could not predict sudden direction changes (i.e., turns) [18]. In general, inaccurate prediction of random moving query leads to frequent updating of an object's location (i.e., communication is an expensive operation). Therefore, reducing the number of location updates is necessary since users do not need to inform the server of their location as long as they follow the predicted path which is known to both server and client.

Researchers have also proposed safe region methods to achieve an efficient evaluation of moving mobile range queries by reducing the communication and updating costs. Some of these methods apply time-based techniques [11–13], while others employ distance-based techniques [2–4]. However, neither time-based nor distance-based techniques are accurate in obtaining a correct query response when they use a fixed time or a fixed distance frame for constructing the safe region. In response, some distance-based techniques use a dynamic distance for constructing a safe region, which is accurate in obtaining the response, but these techniques do not predict when and where the mobile device will leave the safe region.

In this paper, we propose a new method of monitoring moving range query inside the safe region (see Algorithm 2). Our method addresses the problem of knowing when and where the mobile device will leave its safe region, which allows the mobile device to request a new result and a new safe region from the server. In our method the query monitors itself to find out when it will cross the border of the safe region. Using a linear function of time and knowledge of the safe region boundary, our method of monitoring query predicts the query's direction and overcomes the problem of the fork dilemma. Using the new method, if the query makes a sudden turn, the result of the query will not be affected because the query will still be located inside its safe region. Our proposed technique is intended to minimize the costs associated with communication for moving range queries in mobile navigation and to support the concept of random query moves.

## 3. Background

Over the past two decades, spatial database has received increasing interest due to its important role in many modern applications, such as Geographic Information Systems (GIS), multimedia database, navigation systems, urban planning, and traveller information systems [19–25]. The key characteristic that makes this type of databases a powerful tool is its ability to manipulate the data, instead of just storing and representing it. The most basic form of such manipulation is answering queries related to spatial properties of data [26, 27]. The response to a query returns all objects of interest that satisfy the selection conditions and are close to the given query. The most common type of spatial query in spatial and mobile databases is the range search query (static and moving).

### 3.1. Range Search Query

Static range search query, or for simplicity, range search query RSQ, is one of the most frequently used database queries. This type of query is used in Geographical Information Systems (GIS), such as Google Maps, Whereis Maps, Bing Maps, and mobile devices, and also in other applications such as for multimedia database queries [4, 28–33]. RSQ is used to find all objects of interest within a given region or radius and can be defined as follows: given a query point *q* (user's location or query location), a radius *e* (the range of the search specified by the user) and a set of special objects *P* (e.g., hospitals or restaurants) find all objects of interest *P* within radius *e* from *q*.

Many researchers have used range search approaches using an *R*-tree [34] to index multidimensional information due to its efficiency. These approaches typically employ branch-and-bound searching [35] to query spatial points stored in the tree. The *R*-tree is used widely in GIS and is a 2-dimensional extension of the well-known *B*-tree, used for addressing disk. In the case of a *B*-tree, memory is usually partitioned in blocks of some size *B*, on the disk, so that nodes of high degree that fit exactly into one of these blocks and each split are binary. By contrast, an *R*-tree permits multi-way splits arising from internal nodes. This results in a search tree that is shallower than *B*-Tree. The *R*-tree requires fewer blocks from disk when queries follow only one path down the tree, and therefore can be answered more efficiently compared to a binary tree. Usually *R*-tree can answer intersection queries using any query objects (points, line segments, polygons) that can be stored. *R*-tree uses linear storage most of the time in theory, but in worst-case scenarios the query time can also be linear, but this will not make its performance better than a simple linked list [36].

**Figure 1:**
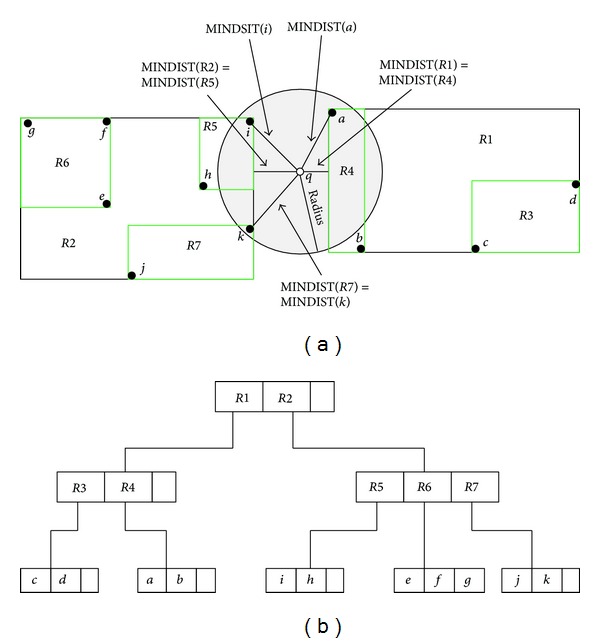
Range search query using *R*-tree index.

Figure 1 illustrates an example which explains the processing of range search using *R*-tree. The range usually corresponds to a circular area or a rectangular window around a query point which is at the centre of the range. All objects whose locations fall within the range area will be counted as objects of interest. Each node has three entries, where the objects {*a*, *b*,…, *k*} represent the set of points. The traversal on *R*-tree starts from the root in a depth first manner, visiting only the internal nodes that have minimum distance (MINDIST) to the query point, which is equal to or less than the radius *e* (e.g, *R*1, *R*2). This process is recursively repeated until all of the leaf nodes that have an equal or smaller distance than *e* from *q* are detected. In this case, nonintersecting entries are pruned because they do not have a qualified point (e.g., *R*3 and *R*6). Also, their minimum distance from *q* is greater than *e*. The step of pruning nonqualified nodes is called a filter step, and the output from the filter step should pass through a refinement step as the object should be examined to specify the result.

### 3.2. Moving Range Search Query

**Figure 2:**
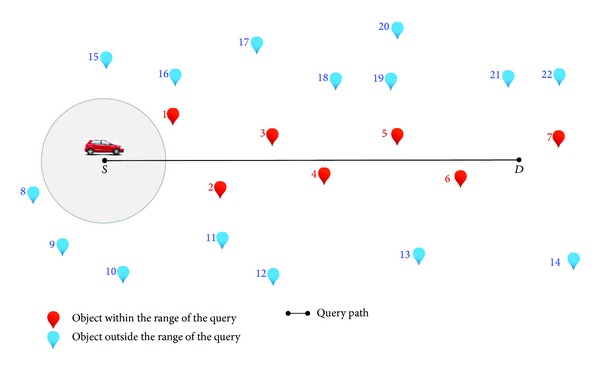
An example of moving range search query in mobile navigation.

A moving range search MRS query is defined as follows: given a set of special objects *P* (e.g., hospitals), a query path *q* = [*S*; *D*] (user's path between two points, *S* = start point and *D* = destination (end) point), and radius *e*, retrieve all objects within the distance *e* to every point in the query path *q* (line segment).  Figure 2 illustrates a moving range search in mobile navigation. The objects of interest (e.g., restaurants) are listed by the numbers 1 to 22. The user wants all objects within 1 km while they are driving (moving) from the start point (*S*) to the destination point (*D*). All of the highlighted objects in red (i.e., the objects 1–7) will be in the result list, while the highlighted objects in blue (i.e., the objects 8–22) will not be included in the result list. The location of the range query needs to be continuously updated whenever the query moves to keep the objects of interest up-to-date.

### 3.3. Safe Region

The cost of monitoring and keeping the location of moving query updated is very high, as the calculation of the range query needs to be reevaluated whenever the query moves to keep the set of objects of interest up-to-date. In response, the concept of a safe region has been proposed. This is an area where the set of objects of interest does not change whilst the query roams inside it.

#### 3.3.1. Basic Safe Region

To construct this type of safe region, the distance between the boundary of the moving query and the closest object to that boundary is calculated (either the object inside or outside the boundary) and this distance is named *e*′. The query has to move this distance (*e*′) before the object enters or leaves the range search (boundary) of this query [2].

**Figure 3:**
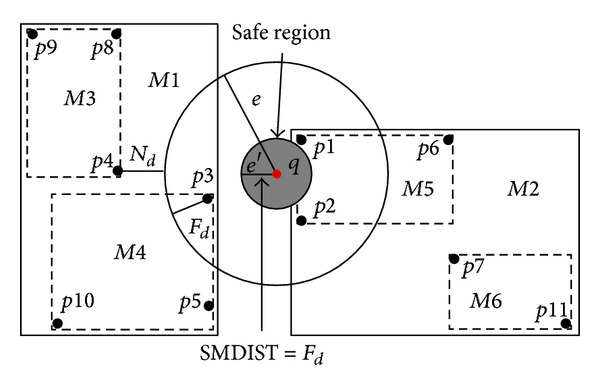
Basic Safe Region.

Drawing a circle around the query within the radius *e*′ represents the basic safe region. Consequently, while the query does not go further than the *e*′ from its original location, the server does not need to check the location of the query because it has not moved outside the safe region.  Figure 3 shows examples of the *basic safe region* in a moving range query. All objects were indexed in *R*-tree [37]. *p*4 represents the nearest object to the boundary of moving query (*q*).

In this scheme, *q* can move in any direction with a distance of *e*′ from its original location without affecting its result; in other words, *q* does not need to update its location while moving within radius *e*′ from its original location.

#### 3.3.2. Extended Safe Region

**Figure 4:**
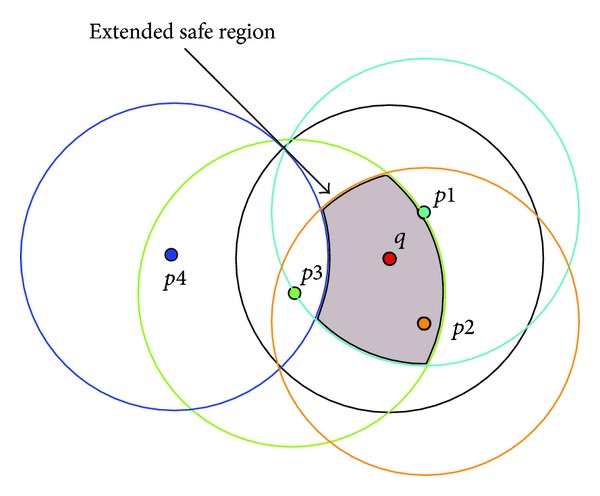
Extended Safe Region.

The objects can be treated as the query, which means that the object is surrounded by a circle equal to the range of the query. Each object within and outside the range boundary is surrounded by a boundary range equal to the range search of the query (see Figure 4). The intersection of these boundaries generates a closed curve containing the query point, representing the extended safe region.

Figure 4 shows an irregular shape containing the query point *q*, representing the Extended Safe Region. The new region is always bigger than the basic safe region [6]; this will give the moving query more space within which to move without informing the server of its new location. By using this technique, the number of communications between the query(s) and the server(s) will be reduced, as well the number of location updates.

## 4. Monitoring the Range Query

This section presents our technique for continuously monitoring a range query inside the extended safe region. A linear function is proposed to monitor the moving query within its safe region. Because the extended safe region has an irregular shape it is necessary to find a method to monitor the query inside it.

### 4.1. Linear Motion Function to Monitor a Query inside Safe Region

This section presents our technique for continuously monitoring a range query inside the extended safe region. A linear function of time model is proposed to monitor the moving query within its extended safe region. Due to the irregular shape of the extended safe region, we need to find a method to monitor the query inside it. This model enables the server to predict when the query leaves the safe region and is based on the query's current location and its velocity. In this technique, we will model the query positions as functions of time. In many circumstances, the area of the safe region is not important; of importance however, is when the query enters and leaves the safe region. In this event the server will be aware of the moving query's location allowing location updates to occur only when the query passes beyond its safe region.

The safe region of the query is computed at the server side based on the intersections of the range objects. The computed safe region will be sent to the moving query from the server. Hence, the query will then send its location to the server when it moves outside the safe region. The query is aware of its current location and its velocity, and in this case the query can calculate its next location(s).

$\bar{x}(t)=({\bar{x}}_{1}(t)$, ${\bar{x}}_{2}(t),\ldots ,{\bar{x}}_{d}(t))$ represents the query's position at time *t*, assuming that the time *t* is not before the current time. In order to model this position, we have used a linear function, which is specified by two parameters. The first parameter represents the position of the object at some specified time *t*_old_, $\bar{x}(t_{\text{old}})$, termed the old position. The second parameter represents the velocity vector of the object, $\bar{v}=(v_{1}$,*v*_2_,…, *v*_3_). Thus, $\bar{x}(t)=\bar{x}(t_{\text{old}})+\bar{v}(t-t_{\text{old}})$. The new position of the moving query is

(1) $$\begin{array}{c} {\bar{x}}_{\text{new}}={\bar{x}}_{\text{old}}+\bar{v}\left(t-t_{\text{old}}\right). \end{array}$$

According to (1), the query knows its position and, hence, there is no need to inform the server about its new location while moving within the safe region. Also, there is no need for the query to inform the server about its velocity when changing its speed and direction.

Generally, the query or the object positions are modelled as functions of time in order to make tentative near-future predictions to alleviate the problem of the frequent updates which will be required. Several studies [18, 38] have used this function to predict the path that the user will use; users may report these parameter values when their actual position deviates from what was previously reported according to some threshold. The prediction of the movement of the object's position can be made from the present into the far future. However, long-term prediction is not possible and short-term prediction suffers from the fork dilemma. Also, it is not usual for a query to exist for a long period of time within a useful threshold of its predicted movement. Therefore, if this query does not report its new position and velocity, after some time, its old positional information will be inaccurate and not useful. Hence, this information will expire.

In our technique, the users do not need to report their parameter values when their actual position deviates from what they have previously reported. Users need to report their new position only when they leave the safe region. After reporting their new position, the server should calculate the new safe region depending on the user's current location and send it to the user. To avoid downlink in the communication and to ensure that the server has received the new user's location, we allocate a time stamp parameter, *t*_stp_, whereby the user should receive a new safe region within *t*_stp_. Our technique does not need any prediction and it overcomes the problem of the fork dilemma. The monitoring of a moving query inside an extended safe region has four scenarios:

1. the query is inside a safe region of one object (the shape of the safe region will be a circle),
2. the query is inside a safe region formed by two overlapping objects, both objects within the result list, (the shape of the safe region has two convex edges),
3. the query is inside a safe region formed by more than two overlapping objects (*n* objects, *n* > 2), but all the objects are within the result list (the shape of the safe region has *n* convex edges),
4. the query is inside a safe region formed by more than one overlapping object (*m* objects, *m* > 1), but some of the objects are within the result list (*n* objects, *n* ≥ 1), and the rest are not within the result list (*r* objects, *r* = *m* − *n* ≥ 1) (the shape of the safe region has *n* convex edge(s) and *r* concave edge(s)).

#### 4.1.1. Query within One Object

**Figure 5:**
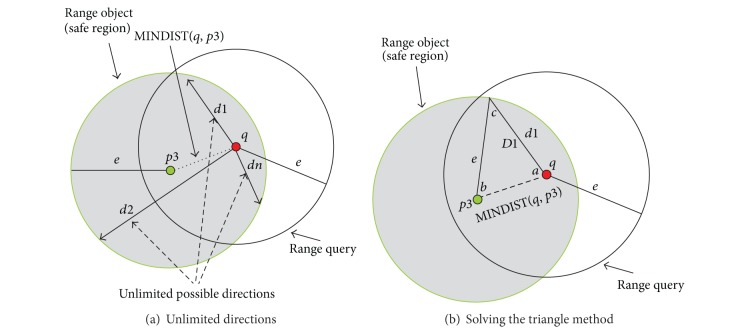
Safe region using linear function.

In some cases, the extended safe region has a circular shape with a range of one object only *pi*. The location of the object *pi* will represent the centre of the extended safe region and *e* will represent the radius. Note that the radius *e* is the range of the query *q*. In Figure 5, the safe region of the query *q* represents the whole range of the object *p*3, where *e*, MINDIST(*q*, *p*3), and the velocity of *q* are known in advance.  Figure 5(a) shows that *q* can move from its current location in any direction (*d*1, *d*2,…, *dn*) depending on its velocity; we will use the triangle solving method to calculate when *q* will leave its safe region.

In Figure 5(b), we consider that *q* will follow the direction *d*1. Using the triangle solving method, we calculate *D*1 (i.e., *D*1 is the distance between the query and the border when the query follows the direction of *d*1) using the triangle shown in this figure. This triangle has the side lengths *D*1, *e*, and MINDIST(*q* · *p*3) and the internal angles *a*, *b*, and *c*. To find *D*1 (the distance when the *q* will be outside its safe region) the angle, *a*, between two sides, MINDIST(*q*, *p*3) and *D*1, should be found first.

The formulas for solving the triangles are

(2) $$\begin{array}{c} c=\arcsin\left(\text{MINDIST}\left(q,p3\right)\times \frac{\sin\left(a\right)}{e}\right). \end{array}$$

The sum of the internal angles of the triangle is 180°

(3) $$\begin{array}{c} a+b+c=180^{\circ }⟶b=180-a-c. \end{array}$$

Now we can find *D*1 by

(4) $$\begin{array}{c} D1=e\times \frac{\sin\left(b\right)}{\sin\left(a\right)}. \end{array}$$

Because the shape of the basic and the Enhanced Safe Region is circular, then this monitoring method can be used to monitor the query in the basic safe region either from the beginning when the query is in the centre of the safe region or when the query changes its direction and it is no longer inside the center of the safe region. This monitoring method can also be used to monitor the query in the Enhanced Safe Region.

#### 4.1.2. Query within Two Objects

**Figure 6:**
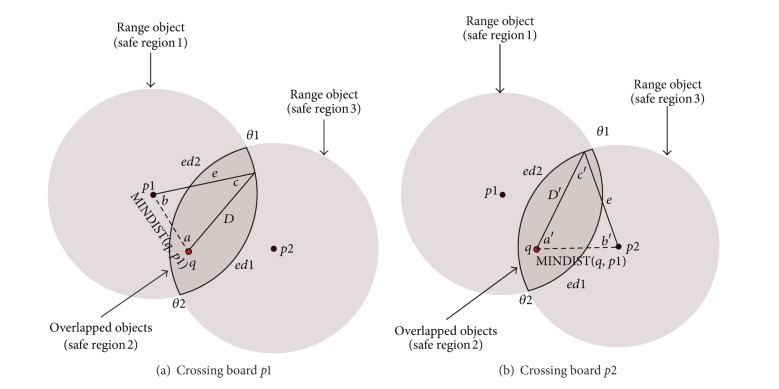
Monitoring *q* within range of two objects.

In the next scenario, the extended safe region might be created by two objects, both of which are within the result list (i.e., both of these two objects are within the range of the query). The overlapping of the ranges of these two objects will create an area which has two edges; both edges (curves) are convex.  Figure 6 shows an example of two overlapping objects (*p*1, *p*2) within the range of the query. Each edge represents the border of the range of one object. The edge within distance *e* from one object *pi* (*pi* ∈ {*p*1, *p*2}) will be the border of that object.

The two convex edges (ed1 and ed2), which determine the Extended Safe Region, are part of the range of the objects (*p*1 and *p*2). The query *q* will be outside the range of an object (*pi*) when it crosses the edge of that object (ed*i*). Crossing any edge of the Extended Safe Region by the query means that the query is leaving its current safe region and entering a new one. If we know in advance the direction of the query, then we can find which edge of the Extended Safe Region the query will cross. To monitor any query in this scenario, the intersection points between the range of the two objects (*p*1 and *p*2) should be found first (see Figure 6). Each curve (edge ed1, ed2) has a start and end angles. For example, the curve ed1 has (*θ*_1_  and  *θ*_2_) as start and end angles, respectively, while the curve ed2 has (*θ*_2_  and  *θ*_1_) as start and end angle respectively.

If the query *q* is moving in a direction within the range of the angle (*θ*_1_ to *θ*_2_), then it will cross the edge of the object *p*1 (ed1) (see Figure 6(a)). If the query *q* is moving in the opposite direction, which is the direction within the range of angle (*θ*_2_ to *θ*_1_), then *q* will cross the edge of the object *p*2 (ed2) see Figure 6(b). Equation (2) can be used to calculate the angle *c*(*c*′). After determining *c*(*c*′), the angle *b*(*b*′) can be found using (3) (i.e., the summation of the internal angles of the triangle, which is 180°). The distance *D*(*D*′) that *q* can move until it reaches the border of the Extended Safe Region can be found using (4).

#### 4.1.3. Query within Multiobjects

Monitoring the query in this scenario is very similar to monitoring the query which was presented in Section 4.1.2. However, we will add the start angle (*θ*_*si*_) and the end angle (*θ*_*ei*_) to each object *pi* surrounding the query. These angles are determined by the intersection of any object with any another one to specify the Extended Safe Region. The start and the end, (*θ*_*si*_) and (*θ*_*ei*_), angles, respectively, identify the corresponding edge of the range object bounding the Extended Safe Region.

**Figure 7:**
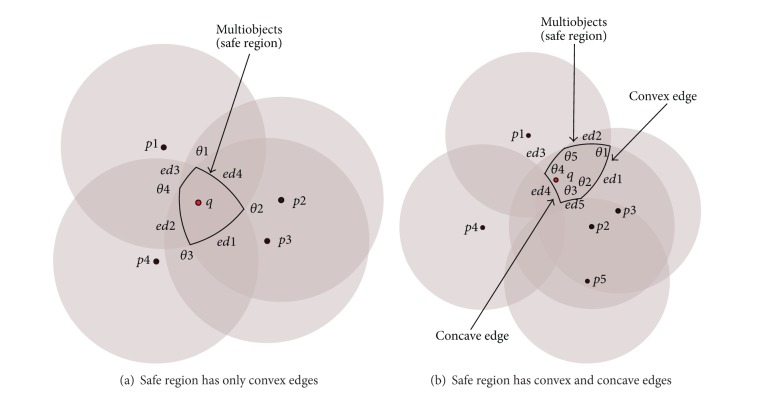
Safe regions with different edges.

The Extended Safe Region will be crossed from a specific edge. It is possible to know this edge based on the velocity of the query (speed and direction) and the starting and ending angles of the object *pi*.  Figure 7(a) shows which edge is crossed by the query when it moves in a specific direction.  Table 1 shows the query will cross *p*1 if it moves at an angle between (110°–200°) and will cross *p*2 if it moves at an angle between (200°–300°) to the rest of the table. Because interior angles must always add up to 360°, (2) can be used to find out the angle *c*, depending on the starting and ending angles of the corresponding edge.

#### 4.1.4. Query within Multiobjects in/out the Result List

In some scenarios, the Extended Safe Region might be created from objects that are inside the result list or outside the result list. Parts of the border of the objects that are inside the result list will be the convex edges of the Extended Safe Region and those objects outside the result list will give the concave edges.  Figure 7(b) shows the convex edge created by the borders of *p*1, *p*2, *p*3, and the concave edge created by the borders *p*4, *p*5. Monitoring the query in this case will occur through the following:

(1) if the query is moving towards the convex edge, then (2) is used to find the angle *c*;
(2) otherwise, the query will move toward the concave edge and then (5) is used. Consider
  (5) $$\begin{array}{c} c=180-\arcsin\left(\text{MINDIST}\left(q,p3\right)\times \frac{\sin\left(a\right)}{e}\right). \end{array}$$

The inverse sine function usually returns angles less than 90°. For example, (2) uses the smallest angle because the third side of the triangle will always be inside the circle (safe region). However, when the third side of the triangle lies outside the safe region the larger angle must be used; see (5). The distance between the query and the object *pi* is used to decide which of (2) or (5) is used. Equation (2) is used when the objects within the range search are within distance *e* from the query, while (5) is used when the objects outside the range of the query are not within the distance *e* from the query.

### 4.2. Support Arbitrary Moving Query

In our moving range query method, the query has the ability to evaluate its location at all times. The query will calculate its current location whenever the velocity of the query changes. By knowing its current location, the query will compute the distance or the time (i.e., when it will leave its safe region and from which edge). The problem of fork dilemma does not occur when the query is inside the safe region because a quick recalculation of the new direction can be made by the query itself and the set of objects of interest does not change.

**Figure 8:**
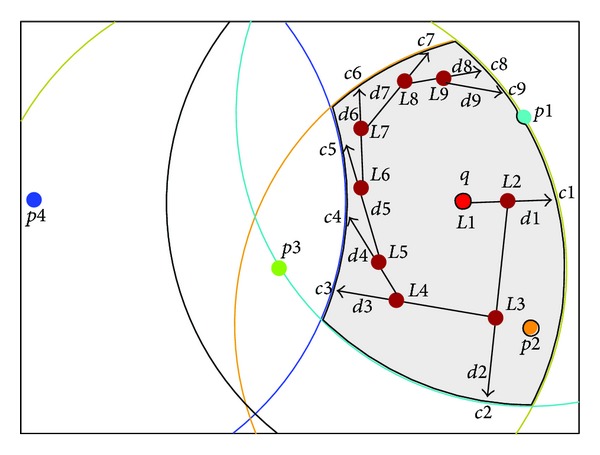
Arbitrary moving query inside safe region.

Figure 8 shows that at each turn the query will calculate its distance to the boundary. The shaded area represents the safe region of the query *q* and the location *L*1 represents its current location. Whenever the query changes its velocity, the query then will compute its new distance to the edge which will be crossed later. This figure shows that the query changes its velocity eight times in order to leave its safe region at the locations (*L*2, *L*3, *L*4, *L*5, *L*6, *L*7, *L*8, *L*9). In each location a new distance from the edge and a new point on the edge (which represents the place that the query will cross the edge from) will be calculated by the query. For example, Figure 8 shows a query moving arbitrarily within a safe region. *L*1 represents the current location of the query, *d*1 is the distance the query can travel before exiting the safe region at point *c*1 unless it changes direction. (*L*2, *d*2, *c*2) represent the new location of the query, the new distance, and the new point on the boundary of the safe region, respectively, and so on with (*L*3, etc.).

## 5. Algorithms

Full details of the Extended Safe Region algorithm and monitoring moving query algorithm are presented below.

### 5.1. Algorithm to Determine the Extended Safe Region

Algorithm 1 calculates the Extended Safe Region of a moving range query. The set of the objects of interest is found first, after which the safe region is calculated. If *pi* is the closest object to the query *q* and MINDIST(*q*, *pi*) ≤ *e*, then any object *pj* will be excluded from the calculation of the safe region if MINDIST(*pi*, *pj*) > 2*e* [2]. Any object that falls above 2*e* from the closest object of interest (*p*1) to the query will not affect the safe region and will be pruned. The objects within distance 2*e* from *p*1 will be surrounded by the circular range of each object having radius *e*. The region formed by the overlap of each of these circular regions containing *q* forms the safe region of *q* at this time. When the query moves outside the safe region, a new safe region should be allocated to the query with the updated result list.

### 5.2. Algorithm to Monitor a Moving Range Query inside Extended Safe Region

An algorithm to monitor a moving query inside an Extended Safe Region is now presented. The safe-object list from Algorithm 1 is used to determine the objects that form the boundary of the safe region. Each object having a shared border with the Extended Safe Region will be registered in a table with the start and end angles of that border. To fill the table of the start and end angles, the safe-object list is constructed first. The list commences with the start and the end angles of the closest border (edge) to the query. The next edge, starting from the end of the previous edge is added to the list. This is continued until the boundary arc terminating at 360° has been added to the list. If the query changes its direction at anytime, then its location will be considered. The new location of the query will be used to find out the distance that the query needs in order to leave its safe region and the point on the boundary at which the query will cross the safe region.

## 6. Experimental Results

Several experiments were conducted to evaluate our proposed algorithms. A synthetic dataset was used to test the proposed linear function. Three different density environments were created (low = 50 objects, medium = 200 objects, and high = 500 objects) to measure the performance of our monitoring method in a data space of 100 km × 100 km.

1000 queries were randomly generated in low, medium, and high density environments. The average distance the query moves until leaving its safe region was then recorded. The average distance from the current location of the query to the border of the current safe region was also recorded in each experiment.

**Figure 9:**
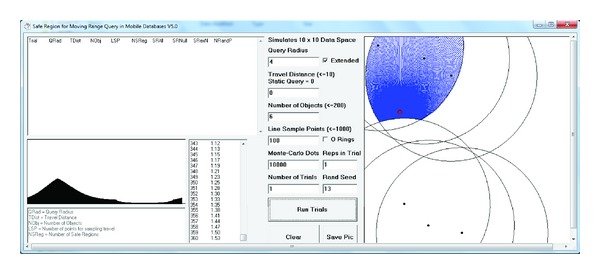
Distance that a query travels before leaving its safe region.

Figure 9 is a screenshot of the software used to calculate the distance that a moving range query can travel before it leaves its safe region when it moves in any direction. This figure shows the distance between the query and the safe region border in all directions. The source of our implementations can be downloaded from the following URL: http://users.monash.edu.au/~khalidi/scientific-world-journal.zip.

### 6.1. Moving Query in Different Environments

**Figure 10:**
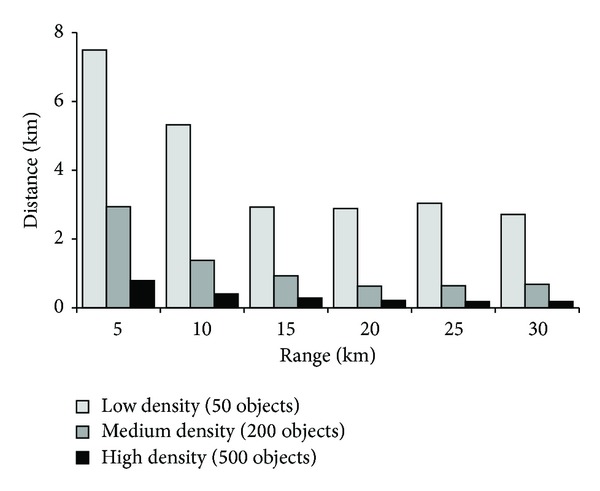
The average distance the query can move until its objects of interest changes in different density environment.

Figure 10 shows a comparison of three different objects environments: low, medium, and high density, with a radius of range search that varies from 5 km to 30 km.

We found that the average distance the query could roam until crossing the border of the safe region is high when there are a few objects surrounding it. The reason is that, in most cases, there is no object within the range of the query and there is a long distance until one object becomes within the range of the query. This gives the query a very good indication of the distance it can travel before the query's set of objects of interest will change or the time until the query will find the first new object when its current list of objects of interest is empty. In range search, when a user invokes a query within a specific range and the result returned is null, the user needs to enlarge the size of the range. However, in our method the user will have an indication about how far the nearest object to the query is and in what direction from the query.

### 6.2. Case Studies

In this section we present two case studies to explain our method for (i) finding when the set of objects of interest will change (when there is already object(s) in the set) and (ii) finding the nearest object in any direction (when there is no object in the result list).

#### 6.2.1. Objects Surrounding the Query

This case study considers a query which has objects of interest in its range. Our linear motion function can inform the query about the distance that the query needs to pass in any direction before its set of objects of interest will change.

**Figure 11:**
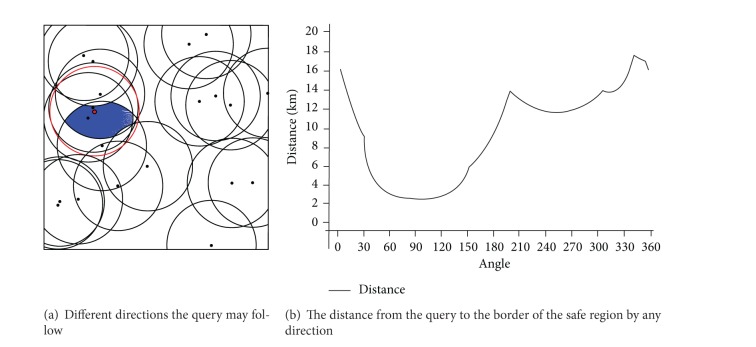
Distance that query can travel before the objects of interest change.

Figure 11 shows a case study of a query with objects surrounding it. In this case the range query is 20 km and the number of objects in the dataset is 20.  Figure 11(a) shows that there are 6 objects surrounding the query in that specific moment (4 objects in the set of objects of interest and two just outside the range).  Figure 11(b) shows the shortest distance (2.3 km) the query can move until the set of objects of interest will change and that occurs when the query moves within an angle of (81°–103°). The longest distance the query can move until the set of objects of interest changes is (17.8 km) at an angle of (343°).

#### 6.2.2. No Object within the Range Query

This case study considers a query which has no object of interest in its range. Our linear motion function can inform the query about the distance that the query needs to pass in any direction before finding the first object.

**Figure 12:**
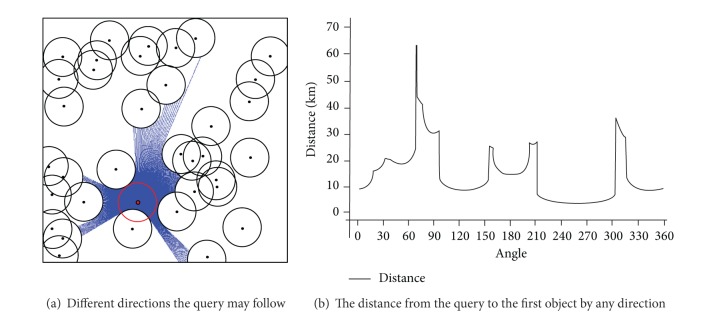
Distance of the closest object in all directions from the query.

Figure 12 shows the distance in any direction to the nearest objects from the query. In this case study the range query is 8 km and the number of objects in the dataset is 35.  Figure 12(a) shows that there is no object within the range of the query at that specific moment.  Figure 12(b) shows that the shortest distance the query can travel to find an object of interest is in the direction (246°–269°) in which case the object will be within 3.1 km from the query, while the longest distance to find an object of interest will be in the direction of the angle (67°) in which case the object will be 64.1 km away from the query.

## 7. Conclusion

We have proposed a linear model to monitor the moving range query inside a safe region for mobile navigation. This approach predicts when the query will leave the safe region based on its current location and velocity. The aim of this technique is to (i) reduce the need for monitoring the query continuously and (ii) to eliminate the need for the user to follow a defined path. The method is used by the query whenever the server allocates a new safe region to it. The method does not suffer from the fork dilemma because it is not calculated as a linear function alone. In contrast, the new method uses time and the concept of the safe region. Hence, if the query makes a sudden turn, the query result will not be affected because the query will still be located inside the safe region.

## Figures and Tables

**Algorithm 1:**
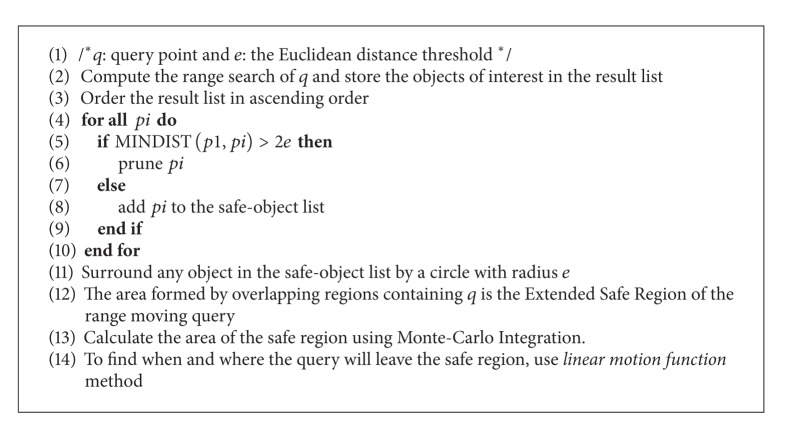
Extended Safe Region algorithm.

**Algorithm 2:**
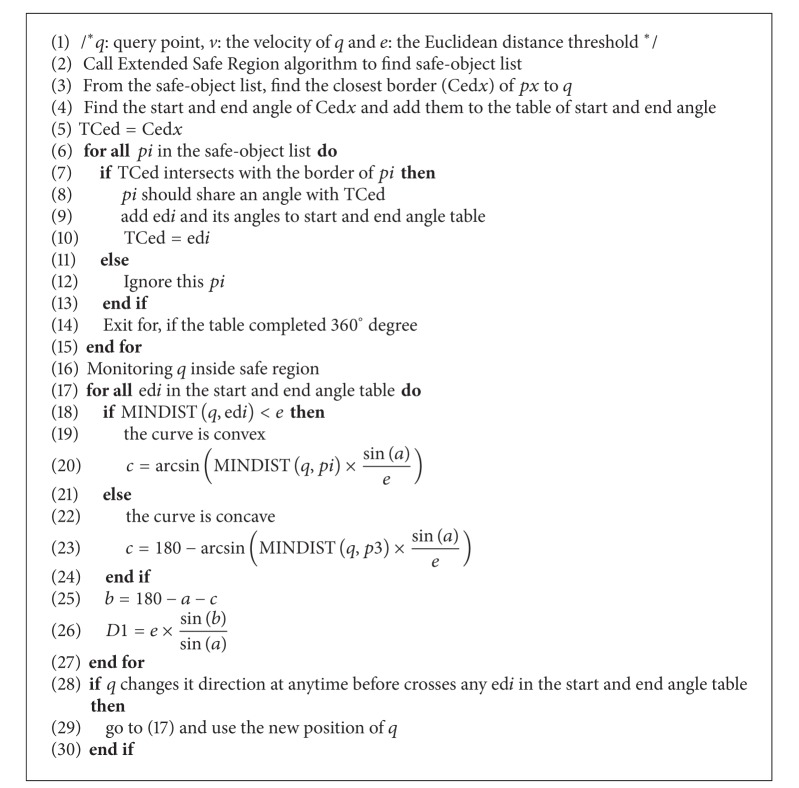
Monitoring moving range query algorithm.

**Table 1:** Start and end angles.

Object	MINDIST(*q*, *pi*)	θ_*si*_	θ_*ei*_
*p*1 (ed1)	1.1	110°	200°
*p*2 (ed2)	1.3	200°	300°
*p*3 (ed3)	1.4	300°	350°
*p*4 (ed4)	1.2	350°	110°
